# Role of Secondary Plant Metabolites on Enteric Methane Mitigation in Ruminants

**DOI:** 10.3389/fvets.2020.00584

**Published:** 2020-08-27

**Authors:** Juan Carlos Ku-Vera, Rafael Jiménez-Ocampo, Sara Stephanie Valencia-Salazar, María Denisse Montoya-Flores, Isabel Cristina Molina-Botero, Jacobo Arango, Carlos Alfredo Gómez-Bravo, Carlos Fernando Aguilar-Pérez, Francisco Javier Solorio-Sánchez

**Affiliations:** ^1^Laboratory of Climate Change and Livestock Production, Department of Animal Nutrition, Faculty of Veterinary Medicine and Animal Science, University of Yucatan, Mérida, Mexico; ^2^National Institute for Forestry, Agriculture and Livestock Research-INIFAP, Experimental Field Valle del Guadiana, Durango, Mexico; ^3^College of the Southern Border (ECOSUR), Livestock and Environment, San Cristóbal de las Casas, Mexico; ^4^National Center for Disciplinary Research in Physiology and Animal Breeding, National Institute for Forestry, Agriculture and Livestock Research-INIFAP, Ajuchitlan, Queretaro, Mexico; ^5^Department of Nutrition, Faculty of Animal Science, National Agrarian University La Molina, Lima, Peru; ^6^International Center for Tropical Agriculture (CIAT), Cali, Colombia

**Keywords:** tannins, saponins, essential oils, flavonoids, methane, ruminants

## Abstract

The rumen microbiome plays a fundamental role in all ruminant species, it is involved in health, nutrient utilization, detoxification, and methane emissions. Methane is a greenhouse gas which is eructated in large volumes by ruminants grazing extensive grasslands in the tropical regions of the world. Enteric methane is the largest contributor to the emissions of greenhouse gases originating from animal agriculture. A large variety of plants containing secondary metabolites [essential oils (terpenoids), tannins, saponins, and flavonoids] have been evaluated as cattle feedstuffs and changes in volatile fatty acid proportions and methane synthesis in the rumen have been assessed. Alterations to the rumen microbiome may lead to changes in diversity, composition, and structure of the methanogen community. Legumes containing condensed tannins such as *Leucaena leucocephala* have shown a good methane mitigating effect when fed at levels of up to 30–35% of ration dry matter in cattle as a result of the effect of condensed tannins on rumen bacteria and methanogens. It has been shown that saponins disrupt the membrane of rumen protozoa, thus decreasing the numbers of both protozoa and methanogenic archaea. Trials carried out with cattle housed in respiration chambers have demonstrated the enteric methane mitigation effect in cattle and sheep of tropical legumes such as *Enterolobium cyclocarpum* and *Samanea saman* which contain saponins. Essential oils are volatile constituents of terpenoid or non-terpenoid origin which impair energy metabolism of archaea and have shown reductions of up to 26% in enteric methane emissions in ruminants. There is emerging evidence showing the potential of flavonoids as methane mitigating compounds, but more work is required *in vivo* to confirm preliminary findings. From the information hereby presented, it is clear that plant secondary metabolites can be a rational approach to modulate the rumen microbiome and modify its function, some species of rumen microbes improve protein and fiber degradation and reduce feed energy loss as methane in ruminants fed tropical plant species.

## Introduction

A rise in the demand of beef and milk in a time horizon to the year 2050 has been predicted due to increasing population, urbanization and the rise in income by some strata of society (1). In the forthcoming years, ruminant production systems will be affected by occasional shocks such as emerging diseases, trade wars, but more regularly by climate change (causing droughts, floods, etc.), which results from the increasing concentrations of greenhouse gases such as carbon dioxide (CO_2_) methane (CH_4_) and nitrous oxide (N_2_O) among other gases in the atmosphere. Ruminant production systems contribute significantly to the emissions of enteric CH_4_ (2) to the atmosphere. Considerable research efforts are being invested in the attempt to mitigate this potent greenhouse gas which is 28 times a higher global warming potential compared to that of CO_2_ (3).

Tropical pastures are characterized by a high concentration of structural carbohydrates (cellulose, hemicellulose) and low concentrations of crude protein (N x 6.25). A fact which constrains anaerobic fermentation of organic matter and induces long retention times of digesta in the reticulo-rumen (4), leading to high emissions of enteric CH_4_ (5). Under those circumstances, it has been claimed that secondary metabolites contained in a wide variety of plants may mitigate CH_4_ emissions through a variety of mechanisms involving the rumen microbiome (6–8). Although secondary metabolites may be usually considered beneficial, the concentration of certain ergot alkaloids such as ergosine and ergocristine in pastures may lead to toxicity under certain circumstances in grazing animals such as sheep and horses (9). Maximal anaerobic fermentation of structural carbohydrates in the rumen is paramount in the transit toward sustainable intensification of tropical animal agriculture by extracting the largest possible amount of useful energy from the rumen which is limited thermodynamically (only 4–5 ATP/mol glucose fermented), for productive purposes. Rumen anaerobiosis dictates that only a limited amount of ATP can be generated from glucose fermentation to volatile fatty acids, heat, CO_2_, and CH_4_ (10). The rumen microbiome is strongly associated to economically and environmental variables which affect feed efficiency and sustainability of ruminant production (11, 12). Increasing the flow of H_2_ in the rumen away from CH_4_ formation toward propionic acid synthesis increase the efficiency of metabolisable energy utilization at the whole animal level while reducing the environmental impact of animal production. It has been recently demonstrated that propionic acid bacteria increase feed degradability and decrease methane production under *in vitro* conditions thus shifting the H_2_ flow away from CH_4_ synthesis toward propionate formation (13), a gluconeogenic precursor.

Other rumen microorganisms are the anaerobic fungal communities, eighteen species from six genera, including monocentric *Neocallimastix, Caecomyces, Piromyces*, and the polycentric *Anaeromyces, Orpinomyces*, and *Cyllamyces*. These groups of microorganisms serve to expose cellulosic components to bacteria to synthesize VFA, but the relationship of fungal abundance with methane emission is still uncertain (14, 15). Rumen metagenome sequencing studies reveal that the higher abundance of Bacteroidetes in the rumen improves feed efficiency, combined with a lower abundance of Firmicutes and methanogenic archaea (16). It has been shown that the reduction of genera such as *Methanobrevibacter* and *Acetobacter* are potential targets for the reduction of CH_4_, on the other hand, an increase in the abundance of the genera *Methanosphaera* and *Eubacterium* led to reductions in CH_4_ emissions in heifers (17). The diversity of the rumen microbiome is related to a myriad of factors such as breed, age, rumen volume and passage rate, geographical location, physiological stage, but essentially to the chemical composition of the ration consumed which is reflected in the resulting pattern of fermentation (15, 18). It is in this context, that plant secondary metabolites (PSM) such as tannins, saponins, essential oils, and flavonoids play an important role in the efforts to mitigate the emissions of CH_4_ from ruminant species. There is a large number of shrub and trees, both legume and non-legume species with great potential for ruminant production in the tropics (19–21) among them, *Leucaena leucocephala* is one of the most promising (22), which contain a wide variety of secondary compounds (23–27) with potential methane-suppressing properties. Secondary metabolites display different mechanisms of action either direct or indirect on the ruminal fermentation and rumen microbiome which decreases CH_4_ synthesis, but an important factor to consider is the persistency of the effect. The aim of the present review is to critically examine the mechanisms of action of secondary compounds contained in a number of tropical plant species on CH_4_ mitigation in ruminants.

## Methane Synthesis

Ruminants are herbivorous that maintain a symbiotic relationship with a large consortium of microorganisms which inhabit the reticulo-rumen. The rumen microbial ecosystem has not been fully studied; therefore, it is difficult to understand all the mechanisms of its functioning, complexity, and interaction among the microbes (28). In the rumen, protozoa, bacteria, and fungi communities ferment enzymatically structural carbohydrates, starch, and proteins. During the fermentation process, volatile fatty acids (VFA), CO_2_, and metabolic H_2_ are produced and used by methanogenic archaea for the synthesis of CH_4_ (7). Methanogenic archaea fluctuate between 10^7^ and 10^9^ cells per milliliter of rumen fluid (29) and approximately two thirds belong to the genus *Methanobrevibacter* and *Methanosarcina* (30) representing 1–4% of the microbial biomass. Protozoa use starch, cellulose, hemicellulose, pectin, and soluble sugars to produce VFA and metabolic H_2_ that is used by the archaea that are attached to its surface to produce CH_4_ (31); thus, there is an association between archaea and the protozoa in the rumen (26, 32). Archaea produce CH_4_ as a metabolic strategy to obtain the energy necessary for their growth (33). Rumen methanogens use the H_2_ resulting from the fermentation of carbohydrates to reduce CO_2_ to CH_4_ in a series of biochemical reactions coupled to ATP synthesis, where CO_2_ is used as a carbon source and H_2_ as the main donor of electrons. In this process, 4 moles of H_2_ are used to produce one mole of CH_4_ (34). The chemical reaction for methane synthesis is CO_2_ + 4H_2_ → CH_4_ + 2H_2_O.

Methanogenesis is the main biochemical pathway for the removal of metabolic hydrogen released from fermentation of carbohydrates in the rumen. A decrease in CH_4_ synthesis would be achieved by inhibiting H_2_-releasing reactions or promoting alternative pathways, where H_2_ is removed during fermentation. The rate of methane production in the rumen depends particularly on the composition of the ration, being the type carbohydrate (cellulose vs. starch), protein and lipids, the components that most influence exert (26, 35), but also on physiological factors such as retention time of digesta in the rumen. The main factor from the ration determining methane synthesis in the rumen is the type of carbohydrate fermented, either structural (cellulose, hemicellulose) typical of forage rations or non-structural (starch, soluble sugars) characteristic of grain (concentrate) rations. The type of carbohydrate being fermented in the rumen also dominates the composition of the rumen microbiome (cellulolytic, amylolytic, pectinolytic, types of bacteria).

Methane is a gas produced in the rumen and is continuously emitted throughout the day; mostly eructated to the atmosphere by the mouth and to a lesser extent by the nostrils (and the anus). CH_4_ has a heat of combustion of 892.6 kJ/mol (i.e., 55.65 MJ/kg) (36) and represents an energy loss (3–12% of gross energy intake) for the animal. There is considerable potential to manipulate the fate of metabolic hydrogen in the rumen, away from methane synthesis toward propionic acid formation which consumes the hydrogen available, by feeding plants containing secondary metabolites to ruminants. Methanogenesis is the main molecular H_2_ sink in the rumen and the partial pressure of H_2_ thermodynamically controls the oxidation-reduction state in this organ for fermentation to proceed for the synthesis of volatile fatty acids, heat, and microbial matter (37). Propionic acid is the only gluconeogenic volatile fatty acid with potential to improve the efficiency of utilization of metabolisable energy in the whole animal for productive purposes (38). The challenge ahead for the development of low-emission animal production systems lies in inducing changes in the rumen microbiome by feeding plants containing secondary metabolites, which will induce alterations in rumen fermentation, rechannelling H_2_ into more energetically efficient biochemical pathways (i.e., VFA synthesis: propionate) which will concomitantly decrease CH_4_ formation.

## Plant Secondary Metabolites for the Reduction of Methane Synthesis

PSM have long been considered important for their protective role against plant predators, their synthesis is regulated by environmental, seasonal, or external stimuli. For years, secondary metabolites have been considered toxic to animals and they were termed anti-nutritional factors (39). However, in the last few decades those metabolites have gained growing interest in animal nutrition due to their beneficial effect for the control of parasites, rumen fermentation, and methane synthesis reduction.

PSM possess ample biological activity in ruminal fermentation processes involved in herbivory and also by their potential to affect growth rate of the rumen microbial population so as to provoke changes that induce mitigation of enteric CH_4_ emissions in ruminants (27). Tannins (8, 40), saponins (25, 41) essential oils (24, 42), and flavonoids (41, 43) have all been evaluated in their potential for enteric CH_4_ mitigation in ruminants. Frequently, their effects on the rumen microbial population are indirect rather than direct. Plants may have a wide variety of secondary metabolites in large or small quantities which may determine their effect on rumen microorganisms. Table 1 describes the effect of PSM on methane production and Table 2 show the effect of secondary metabolites on the microbial population.

**Table 1:** Effect of plant species or plant extracts containing secondary metabolites on enteric methane mitigation in ruminants as measured in open-circuit respiration chambers in tropical regions.

**References**	**Scientific name**	**Family**	**Part of plant**	**Tannins**	**Saponins**	**Essential oils**	**Flavonoids**	**Methane mitigation (%) over control ration**
**Secondary compounds**								
(44)	*Leucaena leucocephala*	*Fabaceae*	Forage	+				20
(45)	*Samanea saman*	*Fabaceae*	Pods	+	+			50
(46)	*Enterolobium cyclocarpum + Gliricidia sepium*	*Fabaceae*	Pods + forage	+	+			6.3
(47)	*Fagopyrum esculentum*	*Polygonaceae*	Rutin				+	0
(48)	*Leucaena leucocephala*	*Fabaceae*	Forage	+				14
(49)	*Termalia chebula*	*Combretaceae*	Seed pulp	+				13
(49)	*Allium sativum*	*Amaryllidaceae*	Bulb	+				2.5
(50)	*Mimosa caesalpiniaefolia*	*Fabaceae*	Forage	+				31.2
(50)	*Eucalyptus spp*.	*Myrtaceae*	Oil			+		30

**Table 2:** Effect of secondary metabolites on rumen microorganisms.

**References**	**Source**	**Study**	**Secondary compound**	**Microorganisms affected**
(51)	Mangosteen peel	*In vivo*	Condensed tannins; Saponins	↑Total baceteria; ↓Methanogens; ↓*R. flavefaciens; =F. succinogenes; = R. albus*
(52)	*Samanea saman*	*In vivo*	Condensed tannins; Saponins	↑*F. succinogene*s, ↓Protozoa; ↓Methanogens
(50)	*Arachis pintoi*	*In vitro*	Condensed tannins	↓Fungi; ↑Methanogens; ↓*R. flavefaciens;* ↓*F. succinogenes*
(50)	*Crotalaria juncea*	*In vitro*	Condensed tannins	↓Fungi; ↑Methanogens; ↓*R. flavefaciens;* ↑*F. succinogenes*
(50)	*Cajanus cajan*	*In vitro*	Condensed tannins	↓Fungi; ↑Methanogens; ↓*R. flavefaciens;* ↓*F. succinogenes*
(50)	*Dolichos labla*	*In vitro*	Condensed tannins	↓Fungi; ↓Methanogens; ↓*R. flavefaciens;* ↓*F. succinogenes;*
(50)	*Leucaena leucocephala*	*In vitro*	Condensed tannins	↓Fungi; ↑Methanogens; ↓*R. flavefaciens;* ↓*F. succinogenes*
(50)	*Mucuna pruriens*	*In vitro*	Condensed tannins	↓Fungi; ↑Methanogens; ↓*R. flavefaciens;* ↓*F. succinogenes*
(50)	*Mucuna aterrimum*	*In vitro*	Condensed tannins	↑Fungi; ↑Methanogens; ↓*R. flavefaciens;* ↓*F. succinogenes*
(50)	*Mimosa caesalpiniaefolia*	*In vitro*	Condensed tannins	↓Fungi; ↑Methanogens; ↓*R. flavefaciens;* ↓*F. succinogenes*
(50)	*Tephrosia candida*	*In vitro*	Condensed tannins	↑Fungi; ↑Methanogens; ↓*R. flavefaciens;* ↓*F. succinogenes*
(53)	*Citrus aurantium; Citrus paradisi* (Commercial product)	*In vitro*	Flavonoids	↓Hydrogenotrophic methanogenic archaea; ↓*Methanosarcina* spp; ↑*M. elsdenii*
(54)	Pomegranate	*In vivo*	Flavonoids and saponins	↑Total protozoal population, ↑*Entodinium* sp. ↑*Isotricha* sp.
(55)	Not specified	*In vitro*	Flavonoids	↑Total bacteria; ↑Protozoa
(56)	Not specified	*In vitro*	Flavonoids	↑Population of general bacteria; > general fungi; ↑*Fibrobacter succinogenes;* ↑*Ruminococcus albus;* ↑*Ruminococcus flavefaciens*
(57)	*Punica granatum, Betula schmidtii, Ginkgo biloba, Camellia japonica, and Cudrania tricuspidata*	*In vitro*	Flavonoids	↓*F. succinogenes;* ↑*Ruminoccocus albus and* ↑*R. flavefaciens*
(58)	*Psidium guajava* leaves	*In vitro*	Flavonoids	= Protozoal count
(59)	*Piper sarmentosum* leaf powder	*In vivo*	Flavonoids	↑Protozoa; = Bacterial population
(60)	*Glycyrrhiza glabra* roots	*In vitro*	Flavonoids	= Total number of bacteria; = archaea diversity
(61)	*Carica papaya* Leaf	*In vitro*	Flavonoids	↑Total bacteria; ↑Total protozoa, ↑*Butyrivibrio fibrisolvens* and ↑Methanogen population
(62)	clove oil, eucalyptus oil, garlic oil, origanum oil.	*In vitro*	Essential oils	↑Archaea, protozoa; ↑*Fibrobacter succinogenes;* ↑*Ruminococcus flavefaciens*, and ↑*R. albus*
(42)	*Thymus capitatus L., Rosmarinus officinalis L., Cinnamomum zeylanicum, Anethum graveolens L.*, and *Eucalyptus globulus Labill and combinations*	*In vitro*	Essential oils	↓*Prevotella* spp., ↓Archaea and ↓Protozoa
(63)	Commercial blend oil	*In vivo*	Essential oils	=Total viable bacteria and protozoa; ↑Cellulolytic bacteria; ↓Hyper ammonia producing bacteria
(64)	*Origanum vulgare* L.	*In vivo*	Essential oils	↑Ruminal fungi; ↓Protozoa; ↑*R. flavefaciens, R. albus* and *F. succinogenes*
(65)	*Cinnamomum zeylanicum* and *Thymus vulgaris*	*In vivo*	Essential oils	↓Methanogens and protozoa; ↓*Fibrobacter succinogenes;* ↓*Ruminococcus albus;* =*Ruminococcus flavefaciens*

## Tannins

**Figure 1:**
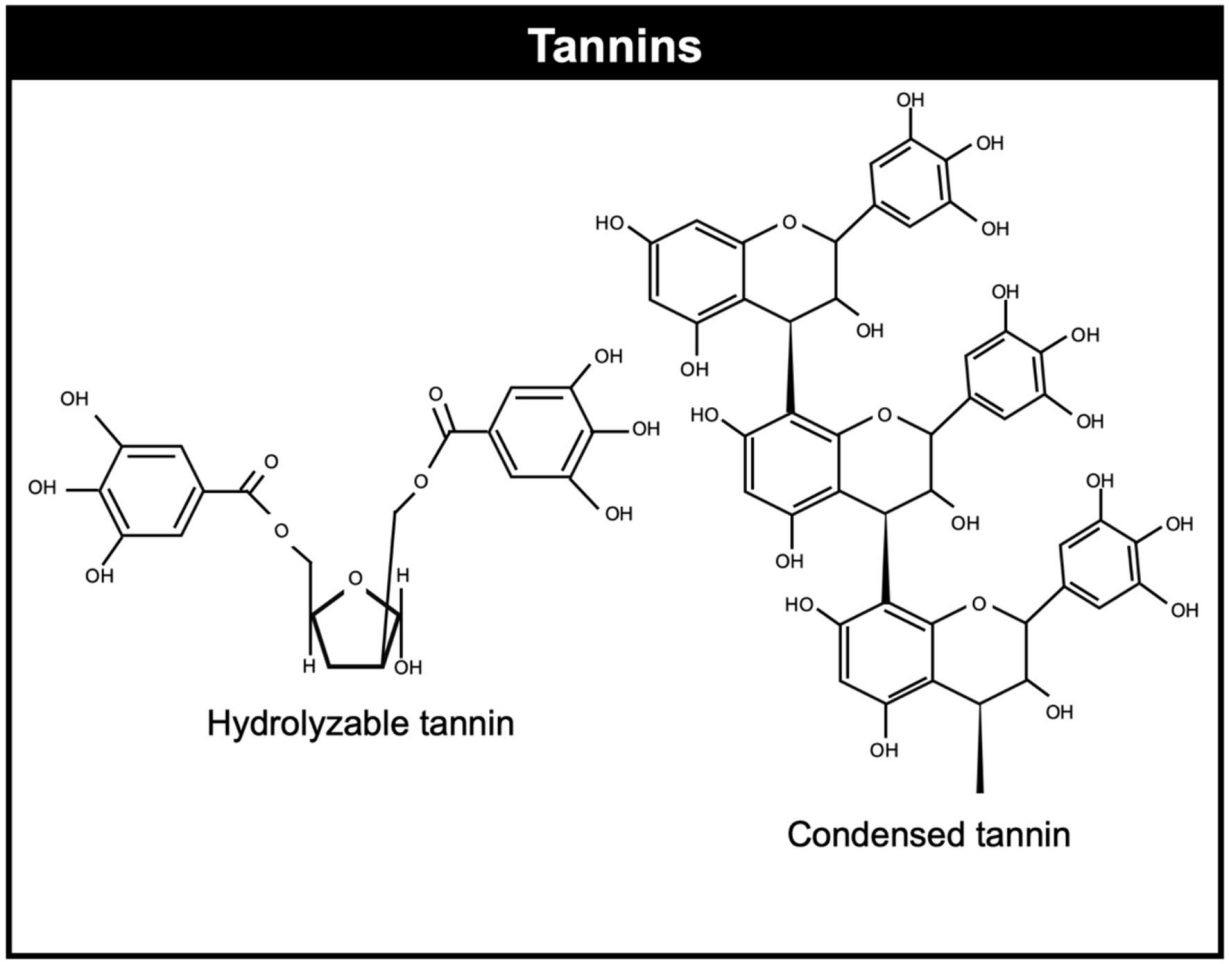
Chemical structure of condensed and hydrolyzable tannins.

Tannins belong a subclass of plant polyphenols (8). Tannins can be divided in hydrolysable and condensed tannins which have different chemical structures (66). Hydrolysable tannins usually contain a polyol core molecule, usually glucose, but also other core molecules such as: glucitol, hammamelose, shikimic acid, quinic acid, and quercitol. On the other hand, condensed tannins distinguish themselves from other polyphenols by their capacity to form complexes and precipitate proteins. They are proanthocyanidins consist of oligomers or polymers of flavan-3-ol subunits (67). Condensed tannins are the secondary metabolites more studied in terms of methane mitigation compared to hydrolysable tannins. Figure 1 illustrates the chemical structure of common condensed and hydrolysable tannins.

Tannins have the capacity to reduce methane synthesis in the rumen directly or indirectly by either inhibiting methanogens or protozoal population, respectively. There are several possible hypotheses to explain the mechanisms of action of tannins on enteric CH_4_ mitigation (40). One of them suggest a direct effect of condensed tannins on rumen methanogenic archaea by binding the proteinaceous adhesin or parts of the cell envelope, thus impairing the establishment of the methanogen-protozoa complex and decreasing interspecies hydrogen transfer and inhibition of methanogen growth (68). Another possible explanation is by indirect inhibition by reducing the availability of nutrients (i.e., carbohydrates, amino acids) to rumen microorganisms, tannin–protein complexes are formed at in the rumen and that postruminal pH shifts in the abomasum (pH <3.5) and the small intestine (pH >7) release protein from these complexes, thus making it available for gastric digestion (69), thus reducing digestibility of feed, and impairing the rumen microbial population. A last theory proposes that condensed tannins act as hydrogen sink themselves diminishing their availability for carbon dioxide reduction to methane, implying that 1.2 mol methane is reduced per mol of catechin (i.e., 6 H_2_ atoms per molecule of catechin) (40).

Crossbred cows fed a mixture of a low-quality tropical grass (*Megathhyrsus maximus*) and increasing levels of chopped legume leaves of *Leucaena leucocephala*, and housed in open-circuit respirations chambers decreased methane production (44) as the level of leucaena intake was increased from 0 to 36% of ration DM. *Leucaena leucocephala* is a legume species widely distributed in Latin America and The Caribbean regions, which is commonly fed to cattle by farmers (70). The reduction up to 20% in methane emissions, agrees with previous work carried out with cows fed *Leucaena leucocephala* in Colombia (48) and in grazing cows consuming leucaena pastures in Australia (71). It is likely that under those conditions, condensed tannins in the legume had an effect on the rumen microbial population reflected in methane reduction. It has been considered that high molecular weight CT fractions of *Leucaena leucocephala* have higher protein-binding affinities compared to low molecular weight fractions, therefore the effect may be associated to the binding ability to cell membranes, resulting in the prevention of nutrient transport into the cell and inhibition of microbial growth (72).

Other tropical legumes such as *Desmanthus* spp. have also induced a reduction in enteric CH_4_ emissions in cattle grazing tropical pastures (73). In India, Pal et al. (74) studied a range of tree leaves containing different concentrations of condensed tannins, demonstrating the strong relationship between tannin content in leaves and methane mitigation under *in vitro* conditions. Condensed tannins (17.2%) and saponins (10.9%) from mangonsteen peel offered at 100 g/head/day to swamp buffaloes increased total bacteria population and *R. flavefaciens* while methanogens were decreased (*p* <0.05) (51). Another study reported a reduction in methane production of up to 25% with mangosteen peel and garlic pellet (75). *Acacia cyanophylla* in an *in vitro* study supplemented at 60 and 30% reduced 37.5 and 56.2% CH_4_ production, respectively due to the high content of condensed tannins that reduced archaea (76).

## Saponins

**Figure 2:**
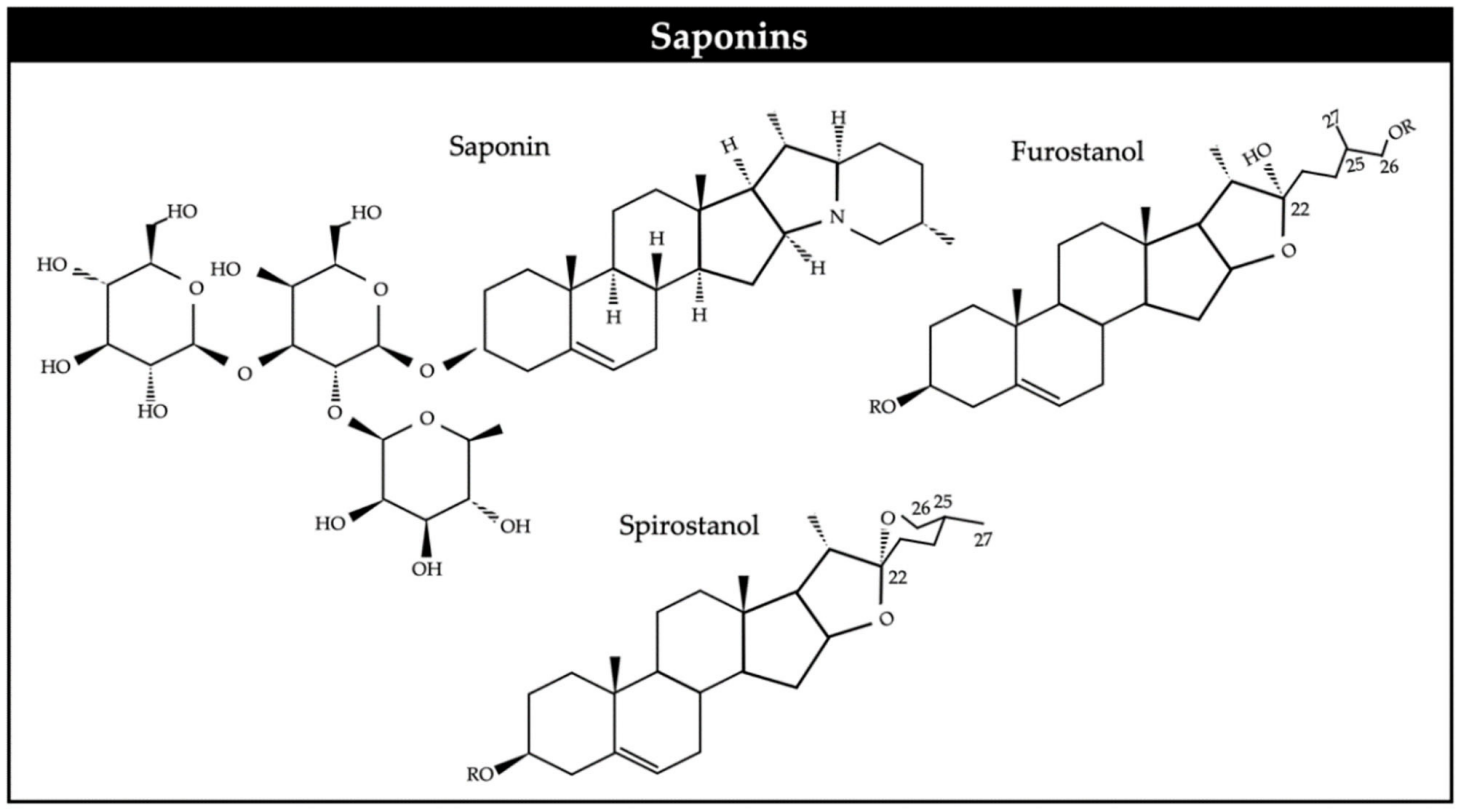
Chemical structure of saponins.

Saponins are high molecular weight glycosides, with a sugar linked to a hydrophobic aglycone (Figure 2). They usually occur as glycosides of steroids or as polycyclic triterpenes. Saponins can be generally classified as steroidal and triterpenoid (77). Saponins are present in a wide variety of tropical trees and shrubs and ruminant species eagerly consume their foliage or pods while browsing. It is generally considered that their main biological effect is on cell membranes. Saponins have been described to be toxic to protozoa (25) and it has been suggested that methanogenic archaea are symbiotically associated to rumen protozoa.

Saponins have the capacity to form complexes with the lipid membrane of bacteria, which increases their permeability, generating an imbalance, and consequently lysis of the microorganism, most of the saponins have an effect on protozoa (78). Wallace et al. (79) proposed that saponins may disrupt protozoa by forming complexes with sterols in the protozoal membrane surface which then becomes impaired and disintegrate. In addition, some saponins have influence on different types of membrane proteins such as Ca^2+^ channels and Na^+^-K^+^ ATPases (80). Ramos-Morales et al. (81) proposed that the effect of saponins on protozoa is temporary due to the fact that bacteria may degrade saponins into sapogenins, a compound that cannot affect protozoa. Wina et al. (82) suggested that protozoa are able to produce extracellular polysaccharides around the membrane to avoid its degradation. In addition, protozoa, depending on the dose and type of the saponin fed, can be reduced in the long term or adapt to the metabolite in the short term (82).

*Samanea saman* (rain tree, in Spanish: algarrobo, genízaro) is a tropical legume present in Mexico, Central America and northern countries of South America as well as Asia and Africa. It contains crude protein and fermentable sugars, the pods fall during the dry season and they are directly consumed by cattle (83). Anantasook et al. (52) supplemented ground pods of *Samanea saman* (rain tree) containing tannins and saponins to rumen-cannulated dairy steers fed a basal ration of urea-treated rice straw and found a reduction in methane emissions also in the protozoal population while propionic acid concentration in the rumen was increased. They concluded that saponins in *Samanea saman* contributed to alter the rumen microbiome by decreasing protozoa and probably methanogenic archaea with the resultant decrease of up to 50% in CH_4_ synthesis. Valencia-Salazar et al. (45) fed crossbred heifers housed in respiration chambers with ground pods of *Samanea saman* at different levels and demonstrated a reduction in enteric CH_4_ production of up to 50% and an increase in propionic acid in rumen liquor, although no effect on the rumen protozoal population was recorded. In this experiment the rechannelling of H_2_ toward propionate synthesis in the rumen, was the most plausible explanation for the decrease in methane emissions.

Foliage and pods (45) of *Samanea saman* show good potential to mitigate enteric methane emissions under practical conditions in farms as part of the ration of grazing ruminants, particularly during the dry season. Similarly, *Enterolobium cyclocarpum* (in Spanish: guanacaste, parota) is a tropical tree which produce foliage and pods which can be employed in ruminant feeding. It is widespread in Central and northern South America and also in Africa (Nigeria). The seeds contain starch (84) and the ground pods of *Enterolobium cyclocarpum* can be readily incorporated in the rations of sheep at levels of 32% (85) and up to 50% (86) with good results in terms of liveweight gain. It has been studied in Canada (87, 88), in Switzerland (89, 90) and in the United Kingdom (91) because of its methane-suppressing properties. It seems the saponins contained in the foliage of *Enterolobium cyclocarpum* affect rumen protozoa population in a selective form (87, 88). The foliage is readily consumed by goats (92). Albores-Moreno et al. (93) found that supplementation of hair sheep with ground pods of *Enterolobium cyclocarpum* (36% DM) decreased enteric methane emissions (estimated by fermentation balance stoichiometry) and the protozoa population, probably as a result of the effect of saponins on the membrane of some protozoa species.

## Essential Oils

**Figure 3:**
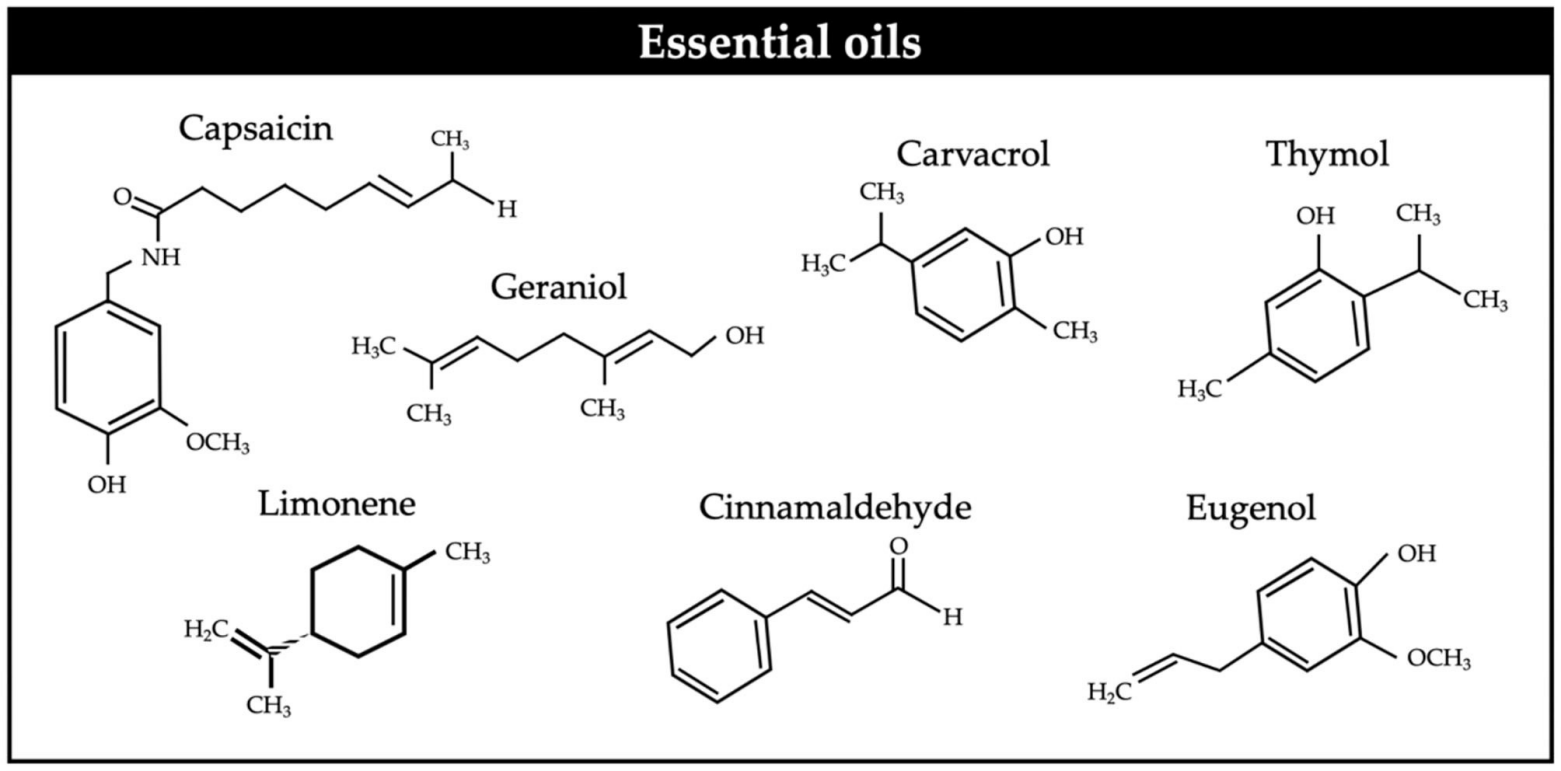
Chemical structures of some essential oils.

Essential oils (EO) are aromatic compounds (Figure 3) largely volatile, which can be found in edible, medicinal, and herbal plants. They are produced in special cells in different parts of the plants, including roots, seeds, fruit, leaves, flowers, bark, petals, and stems (94).

The potential of EO for enteric CH_4_ mitigation has been revised (24, 91). The beneficial effects of EO on the animal such as antioxidant, anti-inflammatory, immune status, and antimicrobial have been shown against a wide variety of microorganisms either Gram-positive or Gram-negative bacteria, fungi, viruses, and protozoa, but more effective against the Gram positive, because most active compounds present in essential oils are lipophilic. In the Gram-negative type, the aromatic hydrocarbons destroy the external membrane (94, 95). This antimicrobial activity is believed to be due to certain terpenoids; (15,000 described in the literature) and phenolic compounds but also to other chemical constituents and functional groups contained in essential oils, 20–60 chemical substances such as acids, alcohols, aldehydes, hydrocarbons, ketones, esters, coumarins, and ethers in trace amounts have been identified (96). EO present a high affinity for the membrane cell of bacteria due to their hydrophobic nature and their affinity for lipids which can disrupt the cytoplasmic membrane either directly or by damaging the membrane proteins, provoking increased membrane permeability, conformational changes, leakage of cytoplasmic components, interfering with bacterial growth and activity, causing changes in the rumen population and fermentation profile (97, 98).

The antimicrobial action has been related to electron transport, ion gradient, protein translocations, phosphorylation, and enzyme reactions (99). It has been proposed that the phenolics thymol and carvacrol inhibit Gram-negative bacteria by disrupting the outer cell membrane and decreasing the concentration gradient (42). EO may cause changes in the archaeal community structure, decreasing methanogen abundance, some protozoa species, and methane production up to 37%, the challenge is finding the appropriate essential oil which minimize the decrease in feed degradation (98). *In vitro* experiments have been encouraging using thymol, carvacrol, cinnamaldehyde, and allicin, limonene and *in vivo* Xtract®6965, rosemary, cinnamaldehyde, anise, garlic, juniper berry, capsicum, ropadiar®, eugenol, crina® blends among others. Mohammed et al. (100) were able to decrease CH_4_ emissions by 19% in steers fed α-cyclodextrin-horseradish oil complex as a methane-suppressing component. However, Benchaar (101) found no effect of oregano oil and carvacrol on enteric CH_4_ emissions when fed (50 mg/kg DM) to dairy cows, Belanche et al. (102) found a decrease in methane emissions when they fed Agolin Ruminant® (essential oils blend) to dairy cows, similarly Castro-Montoya et al. (103) also found a reduction in enteric CH_4_ emissions in dairy cows fed Agolin Ruminant® in the ration during 6 weeks of supplementation.

Wu et al. (104) reported that intermittent feeding of citrus essential oils (d-limonene) has potential to mitigate CH_4_ emissions in Hu sheep by reducing microbial adaptation. Du Han hybrid sheep housed in open-circuit respiration chambers decreased enteric CH_4_ emissions when they were supplemented with essential anise oil, probably because of the effect of the oil on the rumen microbial population (105). It remains to be seen the potential of EO for CH_4_ mitigation under commercial, practical conditions in cattle and sheep farms. Citrus by-products are widely used as energy supplements during the dry season in many tropical countries with good results in animal performance (106, 107). It is of special attention that some essential oils elicit feed consumption due to the aroma they add to the ration and in some other cases negatively affect palatability, as in the case of garlic oil (108). Further trials are needed, to consider the use of essential oils as a commercial option at farm level, learn about the interactions of the active compounds with the ingredients of the ration and their capacity against specific methanogens should be identified without affecting other groups of microorganisms in the rumen, so as not to alter fermentation pattern and rumen degradability, as well as the different doses for each essential oil, the persistence of the enteric CH_4_ mitigating, the possible increase in production and its economic benefits.

## Flavonoids

**Figure 4:**
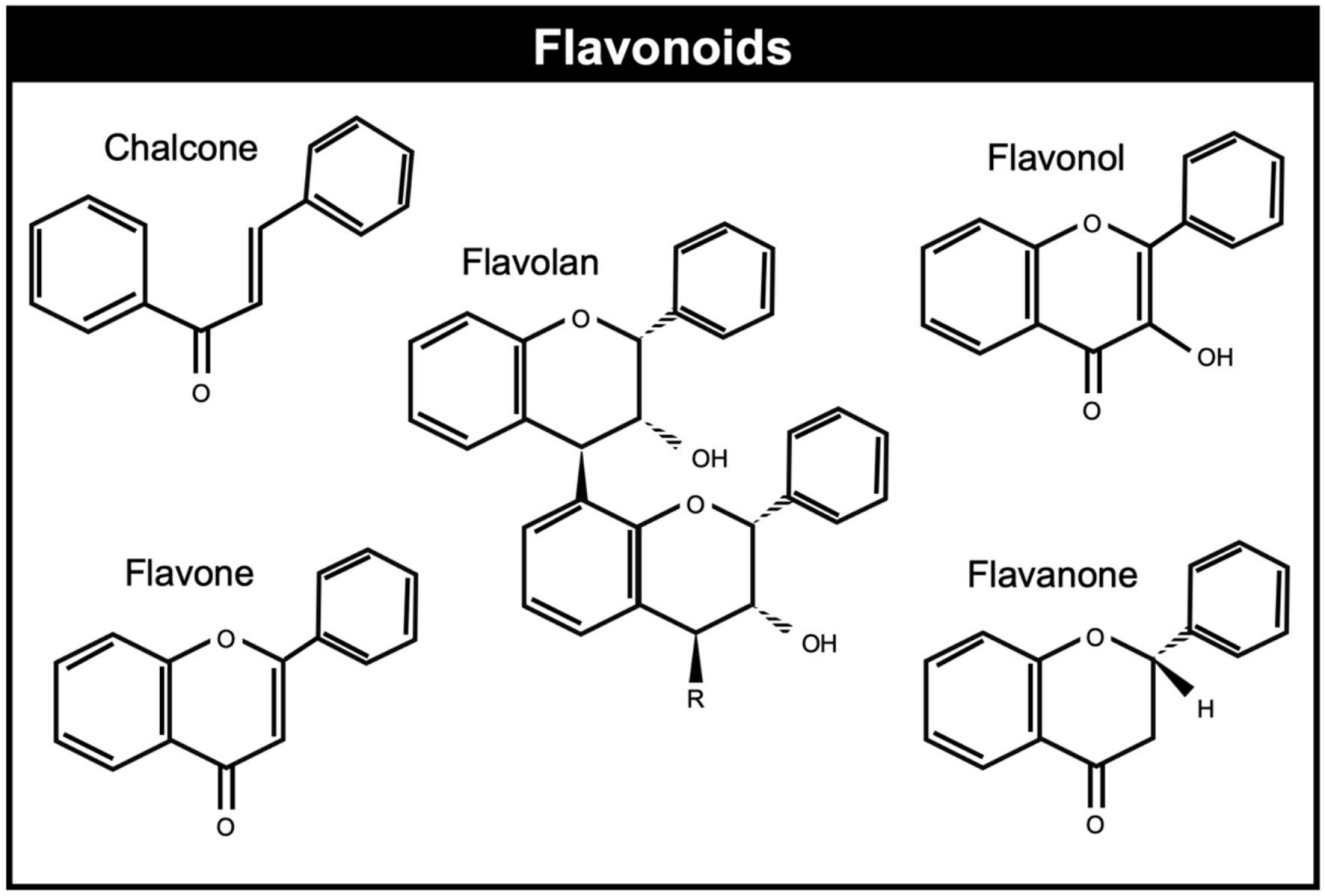
Chemical structures of common flavonoids.

Flavonoids are polyphenols with the C6-C3-C6 skeleton, derivatives of benzo-L-pyrone (109, 110) which are found in seeds and vegetables and display anti-inflammatory, antioxidative, and antimicrobial properties and interfere with different bactericidal factors, including enzymes, toxins, and signal receptors (111) and potential to improve animal welfare. Flavonoids can be generally classified on the basis of their molecular structure and are grouped into eight different flavonoid groups: flavanol, flavandiol, flavanone, dihydroflavonol, flavone, flavonol, isoflavone, and anthocyanidin [(112); Figure 4].

Flavonoids are well-known to be beneficial during periods of animal stress, balance rumen pH in subacute acidosis, reduce the inflammatory state of high grain diets, and for their antibacterial activity which depends on their chemical structure particularly on the substitutions on the aromatic rings (41). Flavonoids exert their effect against Gram-positive microorganisms by inhibiting the function of the cytoplasmic membrane, inhibiting the synthesis of the bacterial cell wall, or by inhibition of nucleic acid synthesis. Flavonoids have been proposed for incorporation in ruminant rations to improve productivity through an increase in the production of propionate relative to acetate (41). Oskoueian et al. (56) reported that the flavonoid naringin and quercetin reduced methane production, ciliate protozoa, and hydrogenotrophic methanogens *in vitro*.

Seradj et al. (53) found that a commercial citrus extract of flavonoids blend (Bioflavex®) reduced methane production, the population of hydrogenotrophic methanogenic archaea and increased the concentration of propionate and the population of *Megasphaera elsdenii in vitro*. Stoldt et al. (47) found that rutin (glucrohamnoside of quercetin) had no effect on methane production or energy metabolism of Holstein cows housed in respiration chambers. Cui et al. (113) reported that the addition of 3.0 mg rutin/kg to diets on multiparous Chinese Holstein cows increased milk yield (10.06 %) in the long term and improved metabolism and digestibility of dairy cows. Other experiments using Holstein cows and supplementation of 60 mg/kg body weight of alfalfa flavonoid extract increased the valeric acid:total volatile fatty acid ratio, the composition of milk, nutrient digestion, and had a tendency to increase the population of *Butyrivibrio fibrisolvens* (114). Recently, Sinz et al. (55) under *in vitro* conditions found that the flavonoid luteolin-7-glucoside did reduce methane. It seems from the data available that flavonoids show potential for methane mitigation but further research in trials carried out *in vivo* are necessary.

The results found with the use of secondary metabolites for the reduction in methane synthesis are variable and dependent on the type of the metabolite, its characteristics and the ration. It is also necessary to verify their effect on methane production in long-term *in vivo* trials due to the possible adaptation of rumen microorganisms to the metabolite and the differences presented between *in vitro* and in *vivo* trials.

## Tropical Plants Containing Secondary Metabolites as a CH_4_ Mitigation Strategy for Smallholders

There are considerable challenges ahead for small livestock keepers in the way toward sustainable intensification of animal agriculture in developing countries (115–117). Enteric CH_4_ emissions originating from ruminant species have been accumulating in the atmosphere from decades ago, with the greatest increase originating from cattle in developing countries, probably because of the rise in animal numbers (118). In the present review, the potential of tropical trees and shrubs (21) to lead the way toward sustainable intensification of ruminant production has been emphasized with data from experiments carried out in developing countries. Some of the important issues to move one step ahead from laboratory experiments to on-farm application of results, are: fair access to markets for smallholders, organization of smallholders (i.e., cooperatives), access to credits and financing, technical support, incorporation of added value to products, development of sustainable value chains (119, 120). Small livestock keepers in developing countries actually use a range of tropical plants (i.e., legumes) to feed their animals on a daily basis, particularly in the dry season and therefore, they are mitigating enteric CH_4_ emissions at present without actually being aware of it. It is responsibility of those in office at agricultural ministries, international agencies (119, 120) or in universities (121) to make this clear to the general public and to decision makers in their areas of influence. It is also important to eliminate the distortions in agricultural policy which unfairly favor economies of scale (large producers) against small livestock keepers. There is great potential to mitigate enteric CH_4_ emissions in the cattle sector in developing countries with a concomitant improvement in productivity and livelihoods of the poor (122–124). Novel approaches in public policies through incentives (tax and trading schemes) for producers applying CH_4_ mitigation practices at farms, may be worth testing in developing countries for widespread adoption (125). Although, it is also highly advisable to improve on the methane inventories of regions or indeed countries from Tier I, to higher levels (i.e., Tier II) of accuracy (126) before any CH_4_ mitigation strategy is put into effect on the field.

## Concluding Remarks

Ruminant production systems will face tremendous challenges in the next forthcoming years due to the increasing demand for beef and milk by the burgeoning population. Climate change (floods, droughts, hail storms) and disease (Covid-19) are imposing a severe negative impact on the health status and economic wellbeing of millions of smallholders in developing countries. Undernutrition is growing rampant in many Latin American countries, particularly in rural areas. Sustainable intensification of ruminant production is a prerequisite to accomplish the ambitious GHG mitigation goals set by national governments to comply with international agreements. There is a need to reduce emissions of greenhouses gases from ruminant production systems while increasing energetic efficiency of protein and fat synthesis in the whole body and in the mammary gland. The ample array of plant secondary metabolites with methane-suppressing potential will have a role to play regarding mitigation of enteric CH_4_ under practical farming conditions in tropical countries with smallholders. Usually, PSM such as tannins, saponins, essential oils, and flavonoids act either by affecting directly methanogenic archaea or indirectly by disrupting the membrane of rumen protozoa. Some secondary metabolites also increase molar proportions of propionic acid in the rumen, thus rechannelling H_2_ away from methanogenesis toward synthesis of propionic acid. In the tropics, supplementation with foliage, pods, tubers, seeds of a range of tropical species seems a rational, and practical approach for enteric methane mitigation under the conditions of small-scale ruminant production systems. Browses and pods of tropical plant species supply rumen fermentable nitrogen for the microbial population, thus improving microbial protein synthesis and degradation of fermentable organic matter in the rumen, a main driver for productivity in ruminant species. As we advance in our understanding of the mechanisms by which secondary metabolites alter rumen fermentation, it is of paramount importance to understand the interplay between the supply of those metabolites, the rumen microbiome and methanogenesis so as to maximize feed efficiency, one of the most important features of financial profitability in ruminant production.

## Author Contributions

JK-V, RJ-O, SV-S, MM-F, and IM-B writing the manuscript. JA and JK-V leaders of the grants. CG-B, FS-S, JA, and CA-P edited the final version of the manuscript. All authors contributed to manuscript revision, intellectual content, and approved the manuscript for publication.

## Conflict of Interest

The authors declare that the research was conducted in the absence of any commercial or financial relationships that could be construed as a potential conflict of interest. The handling Editor declared a past co-authorship with one of the authors JK-V.
